# Current Progress and Future Perspectives of RNA-Based Cancer Vaccines: A 2025 Update

**DOI:** 10.3390/cancers17111882

**Published:** 2025-06-04

**Authors:** Matthias Magoola, Sarfaraz K. Niazi

**Affiliations:** 1 DEI Biopharma, Kampala P.O. Box 35854, Uganda; dei@deigroupinternational.com; 2 College of Pharmacy, University of Illinois, Chicago, IL 60612, USA

**Keywords:** RNA therapeutics, mRNA-based cancer vaccines, circular RNA, nanoparticle delivery, personalized medicine, artificial intelligence, CRISPR integration

## Abstract

RNA-based cancer vaccines have emerged as transformative immunotherapeutic platforms, leveraging advances in mRNA technology and personalized medicine approaches. Recent clinical breakthroughs, particularly the success of mRNA-4157 combined with pembrolizumab in melanoma patients, have demonstrated significant improvements in efficacy, with a 44% reduction in recurrence risk compared to checkpoint inhibitor monotherapy. Breakthrough results from pancreatic cancer vaccines and novel glioblastoma treatments using layered nanoparticle delivery systems mark 2024–2025 as a pivotal period for RNA cancer vaccine development. Current RNA vaccine platforms include conventional mRNA, self-amplifying RNA, trans-amplifying RNA, and emerging circular RNA technologies, with over 120 clinical trials currently underway across various malignancies. Critical advances in delivery optimization include next-generation lipid nanoparticles with tissue-specific targeting and novel nanoengineered systems achieving rapid immune system reprogramming. Manufacturing innovations focus on automated platforms, reducing production timelines from nine weeks to under four weeks for personalized vaccines, while costs remain challenging at over $ 100,000 per patient. Artificial intelligence integration is revolutionizing neoantigen selection through advanced algorithms and CRISPR-enhanced platforms, while regulatory frameworks are evolving with new FDA guidance for therapeutic cancer vaccines. Non-coding RNA applications, including microRNA and long non-coding RNA therapeutics, represent emerging frontiers with potential for enhanced immune modulation. With over 60 candidates in clinical development and the first commercial approvals anticipated by 2029, RNA cancer vaccines are positioned to become cornerstone therapeutics in personalized oncology, offering transformative hope for cancer patients worldwide.

## Simple Summary

RNA-based cancer vaccines are a breakthrough cancer treatment that trains the immune system to recognize and attack specific cancer cells. Recent clinical trials show impressive results, with one melanoma treatment reducing cancer recurrence by 44% when combined with existing immunotherapy. The field gained significant momentum in 2024–2025 with major advances in pancreatic and brain cancer treatments, supported by over 120 ongoing clinical trials. These vaccines utilize different types of RNA to create personalized treatments tailored to each patient’s unique tumor, although they remain expensive, costing over $100,000 per patient. Artificial intelligence is helping scientists identify the best cancer targets while manufacturing improvements have reduced production time from nine weeks to under four weeks. With over 60 treatments in development and first commercial approvals expected by 2029, RNA cancer vaccines represent a significant advancement in personalized cancer care, offering new hope for patients with various types of cancer.

## 1. Introduction

The period from 2024 to 2025 has witnessed unprecedented clinical advances in RNA cancer vaccine development, establishing this therapeutic modality as a viable treatment option across multiple cancer types. The continued success of mRNA-4157 (V940) in combination with pembrolizumab has been reinforced by extended follow-up data, which show sustained clinical benefit, with 3-year recurrence-free survival rates maintaining superiority over pembrolizumab monotherapy [1,2]. This sustained efficacy has catalyzed the global expansion of the Phase 3 trial program, with regulatory submissions anticipated in 2026.

Revolutionary advances in pancreatic cancer vaccines have emerged as a defining achievement of this period. The personalized mRNA vaccine developed by Memorial Sloan Kettering Cancer Center in collaboration with BioNTech demonstrated remarkable efficacy in pancreatic ductal adenocarcinoma patients, with vaccine-induced immune responses persisting for nearly four years after treatment in some patients and showing a reduced risk of cancer recurrence at three-year follow-up compared to non-responders [3]. This breakthrough addresses one of the most challenging malignancies, where only 12% of patients survive five years, representing a paradigm shift for RNA vaccine applications in traditionally immunotherapy-resistant cancers.

The development of novel layered nanoparticle delivery systems has achieved unprecedented rapid immune system activation against brain tumors. University of Florida researchers reported that their mRNA cancer vaccine, utilizing biocompatible lipid nanoparticles with internal fat layers enabling high mRNA loading, successfully reprogrammed the immune system to attack glioblastoma within 48 hours of administration, converting immunologically "cold" tumors to "hot" with vigorous immune cell infiltration [4,5]. This approach demonstrated efficacy across mouse models, pet dogs with naturally occurring brain cancers, and human patients, with treated dogs living nearly four times longer than historical expectations.

Current clinical development encompasses over 120 RNA cancer vaccine trials across various malignancies, including lung, breast, prostate, melanoma, pancreatic, and brain tumors, representing a significant expansion from previous years [6]. The diversity of cancer types under investigation demonstrates the broad applicability of RNA vaccine platforms while emerging data from historically challenging targets validate the transformative potential of this therapeutic approach.

## 2. Advanced RNA Platform Technologies and Novel Delivery Systems

Contemporary RNA cancer vaccine development has evolved beyond conventional mRNA platforms to encompass sophisticated, multi-layered delivery systems and enhanced RNA architectures designed explicitly for cancer applications. The breakthrough layered nanoparticle technology developed at the University of Florida features biocompatible lipid nanoparticles with internal fat layers, enabling the packaging of large numbers of mRNA molecules within each particle to create tumor cells that "look" like dangerous viruses when reinjected into the bloodstream [4].

Circular RNA vaccines continue advancing as promising alternatives for cancer immunotherapy, offering enhanced stability characteristics crucial for prolonged tumor antigen presentation [7,8]. Recent developments have demonstrated that circRNA vaccines retain immunogenic potency after lyophilization and storage at standard refrigeration temperatures, addressing critical cold-chain limitations that restrict the global accessibility of conventional mRNA vaccines [9].

Self-amplifying mRNA platforms are showing particular promise for cancer applications requiring sustained antigen expression and enhanced immunogenicity. These systems incorporate viral replication machinery that enables intracellular amplification of tumor antigen-encoding mRNA, providing prolonged immune stimulation with lower initial doses compared to conventional mRNA approaches [10]. The ability to achieve enhanced potency with reduced dosing requirements offers significant advantages for combination therapy regimens and cost-effective manufacturing.

Engineered nano-vaccines are emerging as a new frontier in cancer therapy, combining traditional vaccine technology with cutting-edge nanoscience to offer superior tumor-targeting precision and long-lasting immune protection. Recent studies have demonstrated that nanoparticle delivery systems can achieve targeted delivery to tumors while reducing side effects [11]. These systems address critical limitations of current delivery platforms by enabling tissue-specific targeting, enhanced tumor penetration, and reduced systemic toxicity.

## 3. Manufacturing Innovation and Personalized Vaccine Production

Significant advances in RNA cancer vaccine manufacturing have addressed key bottlenecks in personalized vaccine production timelines and costs, thereby enhancing the overall efficiency of the process. The manufacturing process for personalized pancreatic cancer vaccines has been optimized to an average of 9 weeks from surgery to the first vaccine dose, with successful vaccine creation achieved for 18 of 19 study participants, representing a substantial improvement in production efficiency [12]. However, manufacturing costs for personalized approaches continue to exceed $100,000 per patient, necessitating continued innovation in automated production systems.

Automated closed-system manufacturing platforms are being implemented to reduce production complexity and human intervention requirements. These systems incorporate continuous processing technologies, real-time quality monitoring, and machine learning-guided optimization to streamline workflows from tumor sample processing through final vaccine formulation. The integration of artificial intelligence in manufacturing enables predictive process control, reducing batch failures and improving consistency across personalized vaccine production [13].

Hybrid manufacturing approaches are gaining traction as practical solutions balancing personalization with scalability. These strategies combine off-the-shelf tumor-associated antigen components with patient-specific neoantigen sequences, enabling the pre-production of common elements while maintaining individualized therapeutic targeting. This modular approach can potentially reduce manufacturing timelines to under 4 weeks while decreasing per-patient costs through economies of scale in shared component production.

Quality control systems have evolved to accommodate the unique requirements of personalized RNA cancer vaccines, incorporating advanced analytical methods to assess mRNA integrity, verify neoantigen sequences, and characterize lipid nanoparticles. These systems must ensure consistent quality across thousands of unique vaccine formulations while maintaining compliance with regulatory requirements for personalized biologics [13,14].

## 4. Artificial Intelligence Integration and CRISPR Enhancement

The integration of artificial intelligence with CRISPR technology represents a transformative advancement in RNA cancer vaccine development, enabling unprecedented precision in the selection of neoantigens and the optimization of immune responses. AI-driven platforms now incorporate multi-omics data analysis to identify optimal tumor-specific targets while predicting immunogenicity and potential immune escape mechanisms [15,16].

CRISPR clinical trials have expanded significantly, with over 100 trials currently active globally. These trials include applications in cancer immunotherapy, where CRISPR-engineered CAR-T cells and gene editing approaches are being combined with RNA vaccine strategies [17,18]. The convergence of CRISPR gene editing with RNA vaccine platforms offers opportunities for enhanced immune system programming, where genetic modifications can optimize T-cell responses to vaccine-delivered tumor antigens.

Machine learning algorithms have achieved remarkable sophistication in neoantigen prioritization, incorporating HLA binding prediction, T-cell receptor recognition modeling, and tumor clonality analysis to select optimal vaccine targets. These systems can process whole-exome sequencing data within hours to generate ranked lists of candidate neoantigens, significantly accelerating the personalized vaccine design process [15,16].

The integration of CRISPR-based editing tools with RNA vaccine production enables real-time optimization of mRNA constructs, including codon optimization, secondary structure modification, and incorporation of immune-enhancing elements. This approach enables rapid iteration and testing of vaccine designs, potentially enhancing immunogenicity and reducing the time required for vaccine optimization [19].

Predictive modeling platforms are incorporating patient-specific immune profiling data to forecast vaccine responses and optimize dosing regimens. These AI systems analyze biomarkers, including tumor mutational burden, immune cell infiltration patterns, and cytokine profiles, to personalize not only vaccine composition but also administration strategies for individual patients [20].

## 5. Regulatory Landscape Evolution and Global Harmonization

The regulatory framework for RNA cancer vaccines has undergone significant evolution, with the FDA’s release of comprehensive guidance for therapeutic cancer vaccines in 2024. The FDA guidance document "Clinical Considerations for Therapeutic Cancer Vaccines" provides sponsors with detailed recommendations for Investigational New Drug applications, addressing critical considerations for both early-phase and late-phase clinical trials specific to cancer vaccine development [21]. This guidance establishes standardized frameworks for clinical trial design, endpoint selection, and regulatory submission requirements.

Experts anticipate that the first commercial mRNA cancer vaccine could receive regulatory approval by 2029, marking a significant achievement in oncology and paving the way for broader applications of mRNA technology in cancer treatment [22]. The regulatory pathway has been accelerated through special designations, including FDA Breakthrough Therapy status and EMA PRIME scheme recognition for leading candidates.

The FDA’s Center for Biologics Evaluation and Research has increased its pace of cell and gene therapy approvals, with eight novel cell and gene therapy approvals in 2024, representing an increase from prior years and signaling the FDA’s readiness to meet its projection of approving 10–20 cell and gene therapies annually by 2025 [23]. This regulatory momentum creates favorable conditions for RNA cancer vaccine approvals as these products advance through Phase 3 trials.

International harmonization efforts have intensified with WHO initiatives to standardize mRNA vaccine manufacturing protocols and regulatory requirements globally. The WHO’s mRNA Technology Transfer Programme continues to expand, supporting the development of manufacturing capabilities in low- and middle-income countries. At the same time, regulatory harmonization frameworks are being established to facilitate global access to approved RNA cancer vaccines [24].

The European Medicines Agency has maintained alignment with FDA approaches while developing EU-specific considerations for RNA therapeutics. The Advanced Therapy Medicinal Products Committee continues providing specialized expertise for novel RNA cancer vaccine evaluations, with several products receiving PRIME designation to expedite the development and regulatory review processes [25].

## 6. Non-Coding RNA Applications and CRISPR-Enhanced Platforms

Beyond conventional mRNA approaches, the integration of non-coding RNA species with CRISPR-enhanced platforms represents a revolutionary advancement in the sophistication of cancer vaccines. MicroRNA therapeutics are being incorporated into vaccine platforms to modulate post-transcriptional gene regulation within tumor microenvironments, targeting immunosuppressive pathways that limit the efficacy of conventional vaccines [26,27].

Specific miRNA candidates, including miR-155 and miR-146a, demonstrate critical roles in dendritic cell maturation and T-cell activation against tumor antigens. The incorporation of these regulatory elements into RNA cancer vaccine platforms creates multi-modal therapeutic approaches that simultaneously deliver tumor antigens while optimizing the immune microenvironment for enhanced anti-tumor responses [26].

Long non-coding RNA applications provide sophisticated transcriptional control mechanisms for enhancing cancer vaccines. Long non-coding RNAs (lncRNAs), such as NEAT1 and MALAT1, influence immune cell differentiation and tumor progression pathways, providing opportunities for targeted interventions that enhance vaccine-induced immunity while suppressing tumor immune evasion mechanisms [27].

CRISPR-based therapies are now integrated into comprehensive RNA therapeutic platforms, with clinical trials expanding rapidly and CRISPR applications showing promise in cancer immunotherapy, where gene editing can enhance immune system targeting of tumor cells [28]. The combination of CRISPR gene editing with RNA vaccine delivery creates opportunities for precisely tailoring immune system reprogramming to individual tumor characteristics.

Small interfering RNA components are being incorporated into cancer vaccine platforms to achieve precise targeting of tumor-associated immunosuppressive factors. These siRNA elements can simultaneously knock down regulatory T-cell pathways, myeloid-derived suppressor cell functions, and inhibitory cytokine production while the mRNA component delivers tumor antigenic stimulation [26,27].

The convergence of multiple RNA species within a single vaccine platform represents a paradigm shift from single-target approaches to comprehensive modulation of the tumor microenvironment. These integrated platforms can address the complexity of cancer immunotherapy by simultaneously targeting multiple pathways involved in immune evasion while delivering robust antigenic stimulation [27].

## 7. Global Access Innovation and Economic Sustainability

The transformation of RNA cancer vaccines into globally accessible therapies requires innovative approaches that address the cost, manufacturing, and distribution challenges currently limiting patient access to these therapies. The NHS Cancer Vaccine Launch Pad, in collaboration with BioNTech, is set to fast-track thousands of patients into trials for personalized mRNA vaccines targeting colorectal, pancreatic, and melanoma cancers, representing a significant advancement in public healthcare system integration of RNA vaccine technologies [29].

Manufacturing decentralization strategies are being implemented to reduce geographic barriers to personalized vaccine access. Regional manufacturing hubs are being established in key markets to reduce shipping timelines and costs while building local technical expertise. These facilities incorporate modular production systems that can be rapidly deployed and scaled based on regional demand patterns [19,24].

Thermostable formulation development continues advancing as a critical enabler for global vaccine distribution. Alternative RNA formats, including circular RNA and lyophilized formulations, are demonstrating enhanced stability at standard refrigeration temperatures, potentially eliminating the need for ultra-cold chains that currently restrict access in resource-limited settings [9,19].

The Asia-Pacific region has experienced the most significant increase in RNA therapy trials, with a growth rate exceeding 26% over the last five years (2019–2023), supported by large patient populations, favorable regulations, and substantial biotech investment [28]. This regional growth creates opportunities for cost-effective clinical development and scale-up of manufacturing.

Innovative pricing models are being explored to enhance global accessibility while maintaining incentives for innovation. These include tiered pricing structures based on national income levels, outcome-based pricing linked to clinical benefit, and volume guarantee mechanisms that provide predictable revenue streams for manufacturers while reducing per-unit costs [19,24].

Technology transfer initiatives are expanding beyond manufacturing to encompass the development of technical expertise, regulatory capability building, and the implementation of quality systems. These comprehensive programs ensure sustainable local production capabilities while maintaining global quality standards for RNA cancer vaccine manufacturing [24].

## 8. Emerging Technologies and Future Integration Strategies

The convergence of multiple advanced technologies is creating unprecedented opportunities for the development of next-generation RNA cancer vaccines. Nanovaccines are emerging as tiny powerhouses—nanoparticles designed to supercharge the immune system, targeting and destroying cancer by merging traditional vaccine technology with cutting-edge nanoscience, thereby offering superior tumor-targeting precision and long-lasting immune protection [11].

Engineered nanoparticle systems are achieving remarkable advances in targeted delivery, with preclinical studies demonstrating enhanced immune cell infiltration into tumors through precise targeting mechanisms. These systems enable the combination delivery of multiple therapeutic agents, including checkpoint inhibitors, cytokines, and tumor antigens within single nanoparticle formulations [11].

The integration of real-time immune monitoring technologies with RNA vaccine platforms enables dynamic treatment optimization based on individual patient responses. Wearable biosensors and liquid biopsy technologies can provide continuous monitoring of immune activation markers, enabling personalized dosing adjustments and optimized combination therapy [20].

Advanced manufacturing technologies, including continuous processing, 3D bioprinting of nanoparticle formulations, and automated quality control systems, are reducing production complexity while improving consistency. These technologies enable distributed manufacturing models where personalized vaccines can be produced closer to patients, reducing logistics complexity and treatment delays [13].

Digital twin technologies are being developed to model individual patient immune responses and predict optimal vaccine formulations. These sophisticated computational models integrate genomic, proteomic, and immune profiling data to simulate vaccine responses and optimize treatment strategies before clinical administration [16,20].

## 9. Clinical Trial Landscape and Regulatory Milestones

The development of mRNA vaccines represents a significant advancement in cancer treatment, with more than 120 clinical trials to date demonstrating their potential across various malignancies, including lung, breast, prostate, melanoma, and more challenging cancers such as pancreatic and brain tumors [6]. This expansive clinical development pipeline represents unprecedented investment and research activity in RNA cancer vaccine development.

Phase 3 trial activity has accelerated significantly, with multiple candidates advancing to pivotal studies. The mRNA-4157 Phase 3 program continues enrollment across global sites while additional candidates in colorectal, lung, and prostate cancers prepare for late-stage clinical development. These pivotal trials will provide definitive evidence for regulatory submissions anticipated between 2026 and 2029 [1,2].

Pediatric clinical development is expanding with dedicated programs for childhood brain cancers and other pediatric malignancies. The University of Florida’s mRNA vaccine for glioblastoma is advancing to expanded Phase 1 clinical trials, which will include up to 24 adult and pediatric patients. Plans are in place for 25 children to participate in Phase 2 studies through partnerships with the Pediatric Neuro-Oncology Consortium [4,5].

Combination therapy trials are increasingly sophisticated, incorporating RNA vaccines with multiple checkpoint inhibitors, targeted therapies, and cellular immunotherapies. These complex trial designs require innovative statistical approaches and adaptive protocols to optimize combination strategies while maintaining feasible enrollment timelines [30].

**Figure 1:**
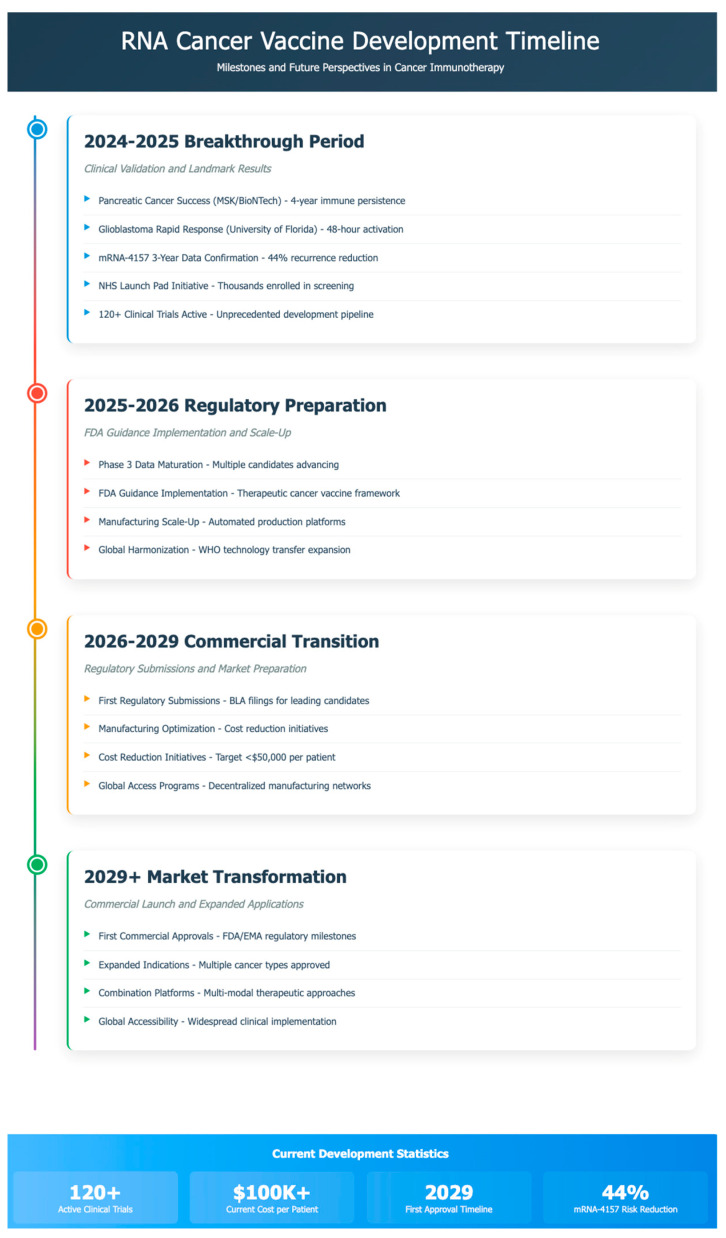
RNA cancer vaccine development timeline and milestones.

Real-world evidence programs are being established to collect post-approval safety and efficacy data across diverse patient populations. These programs will provide critical information on vaccine performance in routine clinical practice, supporting expanded indications and optimized treatment protocols [25] (Table 1 and Table 2). The timeline for the development is portrayed in Figure 1.

## 10. Future Directions and Technological Convergence

The future trajectory of RNA cancer vaccines will be characterized by unprecedented technological convergence, resulting in integrated therapeutic platforms that simultaneously address multiple aspects of cancer immunotherapy. The combination of AI-guided design, CRISPR enhancement, advanced delivery systems, and real-time monitoring technologies will enable truly personalized cancer immunotherapy tailored to individual tumor and immune system characteristics.

Synthetic biology approaches are enabling the design of sophisticated RNA constructs that incorporate multiple functional elements within single platforms. These engineered systems can simultaneously deliver tumor antigens, immune modulators, and genetic regulatory elements to achieve comprehensive reprogramming of anti-tumor immunity [27].

Digital health integration will enable continuous monitoring and optimization of RNA vaccine responses through wearable sensors, liquid biopsy analysis, and AI-powered treatment algorithms. This real-time feedback will allow dynamic treatment adjustments and personalized selection of combination therapy [16,20].

The evolution of manufacturing toward distributed, automated production systems will enable the production of personalized vaccines closer to patients while reducing costs and timelines. These systems will incorporate continuous quality monitoring, predictive maintenance, and adaptive processing to ensure consistent product quality across diverse manufacturing sites [13].

The integration of multiple RNA species within single therapeutic platforms represents the future paradigm for cancer vaccine development. These comprehensive systems will address tumor heterogeneity, immune evasion, and treatment resistance by simultaneously targeting multiple pathways [26,27].

## 11. Conclusions

RNA-based cancer vaccines have achieved unprecedented clinical validation in 2024–2025, with breakthrough results across multiple challenging cancer types, establishing this therapeutic modality as a transformative advancement in oncology. The success of personalized mRNA vaccines in melanoma, pancreatic cancer, and glioblastoma demonstrates their broad applicability and clinical potential. Meanwhile, over 120 active clinical trials represent the most significant coordinated development effort in the history of cancer vaccine research.

Technological innovations, including layered nanoparticle delivery systems, CRISPR-enhanced platforms, and AI-guided design, are creating next-generation therapeutic capabilities that address longstanding limitations in cancer immunotherapy. Manufacturing advances are reducing production timelines while maintaining personalization benefits, though cost challenges remain significant barriers to global accessibility.

Regulatory frameworks have evolved to support accelerated development pathways, with comprehensive FDA guidance and international harmonization efforts creating favorable conditions for clinical advancement. The anticipation of the first commercial approvals by 2029 represents a realistic timeline supported by robust clinical development pipelines and regulatory precedents.

The integration of non-coding RNA applications, CRISPR enhancement technologies, and advanced delivery systems positions RNA cancer vaccines at the forefront of precision oncology. Global access initiatives and manufacturing decentralization strategies are addressing equity concerns while maintaining momentum for innovation.

With continued technological advancement, regulatory support, and international cooperation, RNA cancer vaccines are positioned to become cornerstone therapeutics in personalized oncology, offering transformative treatment options for cancer patients worldwide and ushering in a new era of precision cancer immunotherapy.

## Figures and Tables

**Table 1:** Breakthrough RNA Cancer Vaccine Clinical Results (2024–2025).

Vaccine	Cancer Type	Platform	Key Results	Clinical Status	Reference
mRNA-4157	Melanoma	Personalized mRNA	44% reduction in recurrence risk; 3-year sustained benefit	Phase 3 ongoing	[1,2]
Pancreatic Vaccine	PDAC	Personalized mRNA	4-year immune persistence; reduced recurrence in responders	Phase 1 completed	[3,12]
UF Glioblastoma	Brain Cancer	Layered nanoparticle mRNA	48-h immune activation; 4x survival extension in dogs	Phase 1 expanding	[4,5]
BNT111	Melanoma	Fixed antigen mRNA	Improved response vs historical controls	Phase 2 ongoing	[31]
NHS LaunchPad	Multiple	Personalized mRNA	Thousands enrolled in screening	Phase 2 initiating	[29]

**Table 2:** Advanced RNA platform technologies for cancer applications.

Platform Type	Key Innovation	Advantages	Clinical Stage	Applications
Layered Nanoparticles	Multi-layer fat coating with high mRNA loading	Rapid immune activation (<48 h)	Phase 1	Glioblastoma, solid tumors
Circular RNA	Covalently closed loop structure	Enhanced stability, room temperature storage	Preclinical	Multiple cancer types
Self-Amplifying	Viral replication machinery	Lower doses, sustained expression	Phase 1/2	Hepatocellular carcinoma
CRISPR-Enhanced	Gene editing integration	Optimized immune targeting	Early preclinical	Personalized vaccines
Nanoengineered	Targeted delivery systems	Tumor-specific accumulation	Preclinical/Phase 1	Solid tumors
